# Resolutions and Replies

**Published:** 1908-12-19

**Authors:** 


### Resolutions and Replies.

At the last meeting of the Council of the Royal College of Surgeons the various resolutions which were carried at the meeting of Fellows and Members on November 19 and which were reported in our issue of November 28, were considered, and formal answers to them were given. The first, and perhaps the most important, of these resolutions was that moved by Mr. Joseph Smith and carried almost unanimously, which expressed regret at the Council's action following the poll of Fellows and Members on the question of the admission of women to the College diplomas. To this expression of opinion the Council replied last week that they also regretted that their views, and those of the majority of the members who replied to the circular addressed to them on this subject, were not in accord. By their choice of words the Council thus showed clearly enough that they were impressed and perhaps influenced also by the fact that out of the 13,800 Members (who were all afforded ample opportunities for recording their votes) only 8,543 felt sufficient interest in the matter, or held strong enough views, to fill in their voting papers and return them. While the Council explicitly stated beforehand that they would not necessarily be bound to accept the verdict of the majority in this poll, it is possible that their final decision might have been different if an overwhelming majority of the members had shown themselves opposed to the admission of women to the membership. But the poll indicated, beyond much possibility of mistake, that a large number of Members do not feel very strongly upon the matter either one way or the other, and that the majority are not so violently opposed to the inclusion of women amongst their numbers as to show their feelings when a reasonable opportunity for so doing is afforded them. It is difficult to resist this conclusion when an analysis of the Members' poll demonstrates that, out of the 13,800 who could have voted, 5,257 did not do so at all, 3,920 approved of giving women the right of access to the Conjoint examinations, and only 4,623 (or just over a third of the electorate) expressed disapproval of the proposed measure. Under these circumstances—and in view of the opinion of the Fellows, and of the College of Physicians, and indeed of all the British licensing bodies except Oxford and Cambridge Universities—the decision of the Council to admit women to their examinations cannot with justice be regarded as an outrage upon the views, rights and susceptibilities of the Members, or even as a cynical display of schoolmasterly power and authority. The second resolution of the annual meeting on November 19, proposed by Dr. W. G. Dickinson and carried after some natural dissension, "that women when admitted to the diplomas of the College should have equal rights with men," evoked the answer from the Council that they see no reason for departing from their intention to adhere to that provision of the Medical Act of 1876 which precludes any woman, admitted a Fellow or Member, from taking any part in the government, management or proceedings of the College. This course, which has been decided upon after much careful thought, may strike some people as unjust and paradoxical; but to us, who regard this debateable question as an appendage to the larger issue of Women's Franchise, the Council's decision seems to be wise and far-sighted, and in the best interests of all concerned. The suggestion that the Council should take a further poll of the Fellows and Members on that hardy annual, the direct representation of Members on the College Council, was received with the usual cold courtesy and the usual lack of interest or enthusiasm. With the last resolution, which concerned Conjoint diplomates and the title of "Doctor," and with the Council's reply thereto, we propose to deal in an early issue.

